# Associations between Dietary Patterns, Anthropometric and Cardiometabolic Indices and the Number of MetS Components in Polish Adults with Metabolic Disorders

**DOI:** 10.3390/nu15102237

**Published:** 2023-05-09

**Authors:** Agnieszka Białkowska, Magdalena Górnicka, Monika A. Zielinska-Pukos, Jadwiga Hamulka

**Affiliations:** Department of Human Nutrition, Institute of Human Nutrition Sciences, Warsaw University of Life Sciences (SGGW-WULS), 02-787 Warsaw, Poland; agnieszka_bialkowska@sggw.edu.pl (A.B.); magdalena_gornicka@sggw.edu.pl (M.G.); monika_zielinska_pukos@sggw.edu.pl (M.A.Z.-P.)

**Keywords:** metabolic syndrome, anthropometric data, metabolic dysfunction indices, dietary patterns, food groups, adults

## Abstract

Diet-therapy of metabolic syndrome (MetS) is of great importance due to significant health and social consequences. The aim of this study was (1) to determine dietary patterns (DPs), and (2) to search for associations between defined DPs, anthropometric and cardiometabolic indices, and the number of MetS components in Polish adults with metabolic disorders. The study was designed as a cross-sectional. The study group was 276 adults. Data about the frequency of consumption of selected food groups were collected. Anthropometric measurements: body height (H), body weight (BW), waist (WC), and hip (HC), as well as body composition, were taken. Blood samples were obtained for measurements of glucose and lipids. The obtained biochemical and anthropometric parameters were used to calculate the anthropometric and metabolic dysfunction indices. Three dietary patterns were identified in our study group: Western, Prudent and Low Food. Results of logistic regression analysis indicated rare consumption of fish as a predictor of risk of more severe forms of MetS. The possibility of using body roundness index (BRI) for fast diagnosis of cardiometabolic risk was found. In the management of MetS, the development of strategies to reduce the risk of more severe forms of MetS should be focused on increasing fish consumption and other prohealthy food.

## 1. Introduction

Recently, with the burgeoning global epidemic of obesity and cardiovascular diseases, there is growing concern that the metabolic complications associated with obesity-related non-communicable diseases (NCDs), such as insulin resistance, hypertension, and hyperlipidemia (dyslipidemia) will contribute to many serious public health disorders, which will increase premature mortality worldwide [1]. Metabolic Syndrome (MetS) is increasingly recognized, the pathogenesis of which is very complex and has not yet been fully elucidated. However, in general, MetS can be defined as a cluster of interrelated metabolic factors such as central obesity, impaired glucose tolerance, lipid disorders (elevated triglyceride (TG) levels, low high-density lipoprotein cholesterol (HDL-C) levels and hypertension). 

Central obesity, which also indicates visceral adiposity, leads to low-grade chronic systemic inflammation and is associated with an increased risk of developing atherosclerosis and type 2 diabetes and their cardiovascular complications [2,3]. In addition, central obesity measured by waist circumference is strongly correlated with insulin resistance (IR) [4]. Although the IR is the main mechanism of MetS, it is not directly included in the diagnostic criteria, since the repeated measurement of insulin concentration (necessary for this purpose) is difficult to perform in routine clinical practice. Metabolic syndrome is diagnosed if at least three of the above criteria are present out of five criteria [3,5,6].

The results of epidemiological studies confirm the growing problem related to metabolic syndrome and its individual components (score). It is estimated that metabolic syndrome may affect up to 20–35% (in Europe and the US, respectively) of the world’s adult population [7,8]. The problem is very important, because this value may be underestimated as it does not reflect all regions of the world [9,10]. Regufe et al. [11] report that the global prevalence of MetS ranges from 10% to 84%, being influenced by various socio-economic and demographic factors, among which are age and body mass index. The most rapid increase in MetS is seen in the urban population of developing countries. However, most people diagnosed with MetS are in developed countries, which is associated with a sedentary lifestyle, smoking, unhealthy dietary habits and low socio-economic status [10,11,12,13]. 

It is known that lifestyle factors such as diet and physical activity and sleep duration and quality are crucial for the prevention and treatment, but also for development of metabolic syndrome. The results of observational studies indicate potential associations between various dietary patterns and the risk of MetS [3,14]. Based on the results of cross-sectional studies, it can be assumed that a healthy lifestyle, including proper nutrition, is associated with a lower incidence of MetS [14]. 

Although the main therapeutic strategy in the treatment of MetS is the modification of lifestyle, especially eating habits, the most effective nutritional regimen for treatment has not yet been established. It has been shown that dietary modifications, including improving the quality of food, time and frequency of meals, or changes in the structure of macronutrients, such as fats and carbohydrates, improve individual parameters of metabolic syndrome. Moreover, central obesity, as well as disorders in the metabolism of fats and carbohydrates are associated with many lifestyle factors, such as western type diet (predominance of animal products over plant products, ultra-processed food), low consumption of vegetables and fish, and lack or low physical activity [3,8,12,14].

Diet-therapy of metabolic syndrome is of great importance due to significant health and social consequences, both in the short and long term. Therefore, the aim of this study was (1) to determine dietary patterns (DPs) and (2) to evaluate for associations between defined DPs, anthropometric and cardiometabolic indices and the number of MetS components in Polish adults with metabolic disorders.

## 2. Materials and Methods

### 2.1. Study Design and Participants 

The study was designed as a cross-sectional with convenience sampling and it was undertaken between July 2017 and September 2020. The study group was recruited from among the patients treated in the Metabolic Diseases Outpatient Clinic of the Czerniakowski Hospital in Warsaw. Patients came from both the Warsaw agglomeration and other towns from entire territory of Poland. Over 70% of the group had previously been diagnosed with at least 2 MetS diagnostic criteria, most often (54%) excessive body weight and hypertension. They were referred by general practitioners to the Metabolic Disease Outpatient Clinic. Based on their medical history and medications taken, the doctor decided who to participate in the study. The patients were given a clear and detailed explanation of the scope and the aim of the study. The selection of people for the study was voluntary, after the initial medical qualification and by expressing written consent to participate in the study. Anonymity and confidentiality were respected. The study was approved by the Ethics Committee of the Faculty of Human Nutrition and Consumer Science Warsaw University of Life Sciences, Poland, on 11 April 2017 (Resolution No. 04p/2017). The study was conducted according to the guidelines laid down in the Declaration of Helsinki. Before starting the interview, the interviewer explained the purpose of the study. All participants provided their written informed consent to take participation in the study.

From among 450 people initially expressing their willingness to participate in the study, 335 adults aged 20–70 were recruited. The following inclusion criteria were applied: Caucasian, age over 18 years old; first consultation at the outpatient clinic towards the diagnosis of metabolic syndrome; signed informed consent from the participants. The following exclusion criteria were applied: pregnant or breastfeeding; participation in a weight loss therapy or weight fluctuation in the 6 months prior to the current study; a history of any acute chronic diseases (severe hypoglycaemia, diabetic ketoacidosis), and suffering from serious chronic diseases (cancer, renal failure); taking medications that may affect the results of this study.

**Figure 1:**
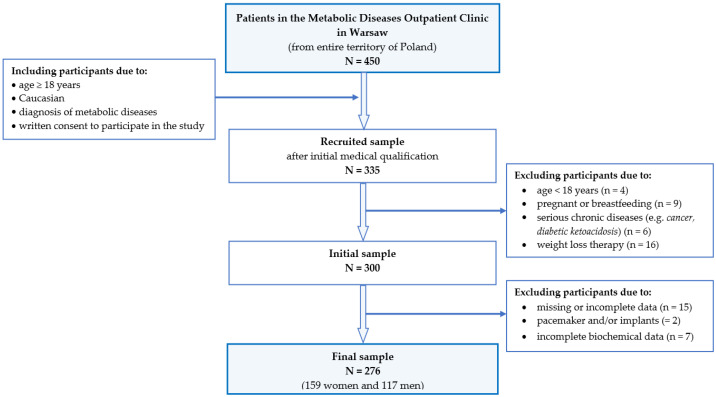
Study design and data collection.

Participants were excluded due to missing or incomplete data, with a pacemaker and/or implants, and if they were unable to draw blood (Figure 1).

### 2.2. Definition and Criteria of Metabolic Syndrome

According to the diagnostic criteria of MetS harmonized in 2009 [5] and the definition of National Cholesterol Education Program Adult Treatment Panel III (NCEP ATP III) [3], MetS is diagnosed if at least three of the five metabolic abnormalities are present (Table 1). Moreover, it was assumed that if the patient is on drug therapy for this component (MetS criterion) he or she is assigned to an abnormal component status.

### 2.3. Data Collection and Procedures

#### 2.3.1. Anthropometrics

Anthropometric parameters: body height (H), body weight (BW), waist (WC), and hip circumference (HC) were measured using standardized procedures according to the International Society for the Advancement of Kinanthropometry (ISAK) International Standards for Anthropometric Assessment guidelines [15,16]. Professional equipment and measuring tape were used. Weight was measured using the electronic digital scale to the nearest 0.1 kg (SECA 799, Hamburg, Germany). Height was measured with the stadiometer with the head in the horizontal Frankfurt plane and recorded with a precision of 0.1 cm (SECA 220, Hamburg, Germany). Waist circumference (WC) was measured with a stretch-resistant tape that provides constant 100 g tension (SECA 201, Hamburg, Germany) at the midway point between the iliac crest and the costal margin (lower rib) on the anterior axillary line in a resting expiratory position. Hip circumference (HC) was measured around the widest part of the buttocks, with the tape parallel to the floor.

Body composition, including fat mass (FM) and fat-free mass (FFM), was assessed using bioelectrical impedance technique using multi-frequency (MF) eight-point Tanita Analyzer (Tanita BC-418 MA, Tanita Co., Tokyo, Japan). Measurements were performed under standardized conditions following to the manufacturer’s protocol: fasting for at least 4 h, avoiding vigorous physical activity at least 12 h prior, not drinking alcohol for 24 h and caffeine for 4 h before the test and urinating before the BIA analysis [17].

All measurements were performed under strictly standardized conditions (room temperature 22 °C, air humidity 45%) by one well-trained researcher (dietitian), using the same device in order to avoid inter-observer and inter-device variability. Measurements were taken twice in light clothing and without shoes and the averages were calculated [16,18].

Adiposity was determined based on three commonly used anthropometric indices: body mass index (BMI > 25 kg/m^2^), waist-to-height ratio (WHtR ≥ 0.5), and fat mass (FM%) according to age and gender (20–39 years, >19% for men and >32% for women; 40–59 years, >21% for men and >33% for women and 60–79 years, >24% for men and >35% for women) [1,6,19]. Moreover, the body roundness index (BRI) was used, calculated according to the formula [18]:

BRI = 364.2 − 365.5 [1 − π^−2^ WC (in m)^2^ × Height (in m)^−2^]^1/2^.

#### 2.3.2. Blood Pressure Measurements

Systolic and diastolic blood pressure (SBP and DBP, respectively) were measured in a sitting position using a SureSigns VM6 Cardiac Monitor (Philips Medical Systems, 3000 Minuteman Road, Andover, MA 01810, USA) on the participant’s right arm in sitting position and after resting for at least 10 min. The measurements were performed twice, according to the National Institute for Health and Care Excellence (NICE) procedures [20] by a single evaluator in a uniform manner for each participant to minimize the bias.

#### 2.3.3. Biochemical Analysis

Blood samples were obtained for measurements of glucose and lipids after overnight fasting (10–12 h), from the ulnar vein between 7 am and 9 am, using standard techniques. Blood was centrifuged for 10 min at 5000 rpm in min at 4 °C and stored frozen (−80 °C) for the further analysis.

All biochemical analyses were determined by a certified laboratory using standard methods. The fasting plasma glucose (FPG) concentration in the blood serum was measured using the enzyme method with hexokinase. The concentration of total cholesterol (CHOL) was determined by means of the enzyme method with esterase and cholesterol oxidase, high-density lipoprotein (HDL-C) and triglyceride (TG), with the use of the colorimetric non-precipitation method. The LDL cholesterol blood level (LDL-C) was calculated using Friedewald formula [21]. 

#### 2.3.4. Metabolic Dysfunction Indices

The obtained biochemical and anthropometric parameters were used to calculate the metabolic dysfunction indices such as: atherogenic index of plasma (AIP), cardiometabolic index (CMI), lipid accumulation product (LAP), triglycerides to HDL-cholesterol ratio (TG/HDL-C), triglycerides–glucose index (TyG), TyG-body mass index (TyG-BMI), TyG-waist circumference index (TyG-WC), visceral adiposity index (VAI) [6,22].

The metabolic dysfunction indices were calculated by the formulas:

AIP = log (TG/HDL-C);

CMI = (TG/HDL) × WHtR;

LAP for females = (WC − 58) × TG (mmol/L);

LAP for males = (WC − 65) × TG (mmol/L);

TyG = Ln[TG(mg/dL) × fasting glucose (mg/dL)/2];

TyG-BMI = TyG × BMI;

TyG-WC = TyG × WC;

VAI for females = WC/(36.58 + (1.89 × BMI)) × TG/0.81 × 1.52/HDL-C;

VAI for males = WC/(39.68 + (1.88 × BMI)) × TG/1.03 × 1.31/HDL-C.

#### 2.3.5. Dietary Assessment

A Dietary Habits and Nutrition Beliefs Questionnaire (KomPAN) [23,24] was used to assess the frequency of consumption of selected food groups such as milk, fermented milk products, cottage cheese, cheese, red meat, white meat, processed meat products, fish, wholegrain products, refined grain products, vegetables, fruits, fried foods, fast food, sweets, juices, sweetened drinks, water, coffee or strong tea and energetic drinks. All participants were asked to record their habitual frequency of consumption for each food group within the last three months according to the following categories: ‘1—never or al-most never’, ‘2— less than once a week 3—once a week’, ‘4—2–4 times a week’, ‘5— 5–6 times a week’, ‘6-once a day’, ‘7—a few times a day’. 

The respondents also provided information about special dieting, consumption of meals at consistent times and the number of meals eaten per day (by choosing one of five answers, ranging from one meal a day to five meals or more a day), and snacking between meals.

#### 2.3.6. Dietary Patterns Identification

Dietary patterns were distinguish using k-means analysis including 18 food groups (milk products: milk, fermented milk products, cottage cheese), fish, juices, water, sweetened drinks, coffee or strong tea, energetic drinks, vegetables, fruits, sweets, wholegrain products, refined grain products, fried foods, cheeses, processed meat products, red meat, white meat, fast food). Three dietary patterns were created: (1) Western, characterized by high consumption of processed foods, red meat, cheese and fried foods; (2) Prudent, characterized by the higher consumption of vegetables, milk products, fish, wholegrain products, water, and the lowest consumption of juices, refined grain products, fried foods, fast foods and red meat; and (3) Low Food, where the low intake of milk products, fish, cheese, coffee and strong tea, vegetables, fruits, sweets, processed meat and white meat were observed (Table S1).

#### 2.3.7. Sociodemographic and Lifestyle Data

The data were collected using a standardized, structured and detailed questionnaire by one well-trained researcher. The collected data included demographic information (age, sex, education, occupation, financial status, etc.), medical history (self-reported illness history and current medications), lifestyle factors (physical activity, smoking and alcohol consumption), and family history of any chronic diseases. Regarding smoking, as former smokers, we defined people who had not smoked for at least three months. We considered people who never smoked or smoked occasionally in their youth non-smokers. Physical activity (PA) was assessed separately for the physical activity during leisure and work/school time. The respondents chose one of three categories describing their physical activity at school and during leisure time: 1—“low”; 2—“moderate”; 3—“vigorous”. Examples for each category of PA were provided to choose from. “Low” physical activity in leisure time was described as “sedentary lifestyle, watching TV, reading the news, books, light housework, taking a walk for 1–2 h a week”; “moderate PA” was defined as “walks, cycling, gymnastics, gardening or other light physical activity performed for 2–3 h a week”, and “vigorous PA” was defined as “cycling, running, working on a plot or garden, and other sports activities requiring physical effort, taking up more than 3 h a week”. Finally, after combining categories of responses, three description PA levels were created: “low PA”—over 70% of the time in a sitting position; “moderate PA”—‘approximately 50% of the time in a sitting position and moving for about 50% of the time; and “vigorous PA”—being in motion for about 70% of the time or doing physical work associated with a lot of effort [23].

#### 2.3.8. Statistical Analysis

Data were presented as a sample percentage (%) for categorical data or mean and standard deviation (SD) for continuous data. The study group was divided according to sex and the number of MetS components (MetS score), assuming a higher risk of a more severe form of MetS for MetS 4 and 5. The differences between groups were verified with the Pearson Chi squared test (categorical data) or the Kruskal–Wallis test with post-hoc analysis (continuous data; for more than two groups) or the U Mann Whitney test (continuous data; for two groups). In addition, the partial correlation between anthropometric indices, cardiometabolic indices with hemodynamic indices, the lipid markers and number of MetS components was investigated with the Spearman correlation test. Before statistical analysis, the normality of variable distribution was checked with the Shapiro-Wilk test. The logistic regression analysis was performed to investigate nutritional factors increasing the risk of more severe form of MetS (MetS score 4–5). Prior to logistic analysis, food frequency categories were recategorized to three categories: (1) never or almost never; (2) at least one time per week; (3) at least one time per day. Three models were calculated: univariate (model 1); multivariate adjusted for sex, age, education status, physical activity and smoking status (model 2); and multivariate adjusted for sex, age, education status, physical activity, smoking status and dietary patterns (model 3). We presented models for frequency of fish, fried foods consumption and dietary patterns (no other food groups were statistically significant). For all tests, *p* ≤ 0.05 was considered significant. The statistical analysis was performed using STATISTICA software version 13.0 (StatSoft Inc., Tulsa, OK, USA; StatSoft. Krakow, Poland).

## 3. Results

### 3.1. Participant Characteristics 

The study group consisted of 276 adults, including 159 women and 117 men. Taking into account the MetS components, it was found that 54% were individuals with three MetS components, 26% with four and 20% with all MetS components (Table 2). The mean age of the participants differed statistically significantly between subgroups, 51.6 years for individuals with three MetS components and 58.2 years for ones with five MetS components. Men were characterized by higher number of MetS components than women. In the subgroup with three MetS, the percentage of individuals with normal body weight was the highest (31% vs. 8.6% and 3.6% for subgroups with four or five MEtS, respectively), and the lowest with class III obesity (8%, vs. 15.7% and 17.9% for subgroups with four or five MetS, respectively). The mean values of BMI, BRI, WC, WHtR, and blood pressure were the lowest in individuals with three MetS components. Among biochemical MetS components, the lowest glucose and TG concentration, as well as the highest HDL-C concentration were found in three MetS sub-group. Significant differences were found between the MetS subgroups for all used cardiometabolic indices. However, there were no significant differences in dietary patterns depending on the number of MetS components.

### 3.2. Dietary Patterns and Frequency of Consumption of Selected Group Products 

Dietary patterns and selected group products according to the number of MetS components are presented in Table 3. The prevalence of the created DPs did not differ in the group of men from the number of MetS components. While the tendency was a domination of the Western DP in women with four MetS components, Prudent in three MetS and Low Food in five MetS were found. The average frequency of pro-healthy products intake was as follows: fruit, vegetable and water—once a day; milk, fermented milk beverages, cottage cheese—several times a week; fish—almost once a week; white meat and whole grains products—2–4 times a week (Table 3). Meanwhile, the non-recommended products such as processed meat or non-whole refined grains were consumed almost once a day; red meat, sweets and fried food—2–4 times a week; sweet beverages—1–4 times a week. 

In men, significant differences were found in the average frequency of consumption only for milk, fermented milk drinks and cottage cheese. Men with three MetS components consumed them 5–6 times a week, and men with five MetS components 2–4 times a week. In women, sweets were more often consumed in three or four MetS subgroups, while non-whole refined grains products in four or five MetS sub-groups. In three MetS sub-group, men consumed fried and fast food more often than women, and in five MetS sub-group men consumed sweet beverages more frequently than women. There was a tendency for more frequent consumption of cheese in men with three MetS, milk and milk products by women with four MetS, and fruit and fried foods by women with five MetS when compared to the corresponding MetS subgroups.

### 3.3. Anthropometric Parameters, Cardiometabolic Indices and Number of MetS Components

Anthropometric parameters, MetS components and cardiometabolic indices according to sex and number of MetS components are presented in Table 4. Regardless of sex, the values of anthropometric parameters, systolic blood pressure, fasting glucose, triglycerides and cardiometabolic indices differed significantly and the highest values were observed in sub-groups with five MetS components. Higher HDL-C concentrations were found in both men and women with three Met components. The values of BRI and WHtR in women with five MetS components were significantly higher than in men (Table 4). The percentage of fat mass was higher in women and it differs significantly in each MetS subgroup, reaching values in the range 23.2–29.2% for men and 36.9–42.1% for women. Similar effects (dependencies) were found for the VAI index, which was in the range 2.2–4.4 for men, and 2.8–5.4 for women. Taking into account the lipid profile, only the concentration of HDL-C in the plasma was significantly higher in women from sub-groups with three and four MetS components compared to men from the corresponding subgroups.

The partial coefficients of Spearman correlation of the anthropometric indices, cardiometabolic indices with the lipid markers and number of MetS components are shown in Table 5. The strongest positive correlation (r > 0.8) was found between BMI and: WHtR (0.900, *p* < 0.001), BRI (0.899, *p* < 0.001), WC (0.889, *p* < 0.001), %FM (0.830, *p* < 0.001); BRI and: WHtR (0.995, *p* < 0.001), WC (0.958, *p* < 0.001). %FM was positively correlated with BMI (0.830, *p* < 0.001), WC (0.804, *p* < 0.001), WHtR (0.802, *p* < 0.001) and BRI (0.789, *p* < 0.001). There was a weak negative correlation between HDL-C concentration and WC, WHtR, BMI and BRI, as well as fasting glucose. For TG weak positive correlation with WC, WHtR, BRI, as well as fasting glucose, was found. All cardiometabolic indices used positively correlated with MetS score, which was also positively correlated with BRI (0.376, *p* < 0.001), the strongest among anthropometric indices being and WHtR and WC (respectively, 0.366 and 0.363, *p* < 0.001). The least positively correlated with the MetS result was WHR (0.214, *p* < 0.01).

### 3.4. Association between MetS Severity and Selected Nutritional Variables

Results of logistic regression analysis are presented in Table 6. Consumption of fried foods at least one time per week and at least one time per day compared to never or almost never intake increased the risk of more severe forms of MetS (*p* ≤ 0.05) by two to three times. However, after adjustment for potential confounders (sex, age, education status, physical activity and smoking status) those results were no longer significant. On the contrary, rare consumption of fish increased the risk of more severe forms of MetS by 35% in the univariate model and nearly two times after adjustment for sex, age, education status, physical activity, and smoking status separately and with consideration dietary pattern. No other food group or dietary patterns were significant predictors of risk of more severe forms of MetS.

## 4. Discussion

Three dietary patterns were identified in our study group: Western, Prudent and Low Food. Their prevalence did not differ in the group of men, while the tendency with domination of Prudent dietary patterns in women with three MetS was found. The frequency of consumption of healthy and unhealthy food was not in accordance with the recommendations. The results of the logistic regression analysis indicated that rare consumption of fish may be a predictor of the risk of a more severe form of MetS. Among anthropometric indices, the strongest correlation between BRI and number of MetS score was found.

MetS is a disease with a complex pathogenesis, involving both genetic and modifiable behavioral factors, such as food intake and physical activity [3,12]. Modifiable factors related to lifestyle are crucial in the prevention of METs, but also at every stage of treatment of METs, especially in early diagnosis. Dietary patterns that include health-promoting food groups such as vegetables, fruits, nuts, legumes, fish and seafood, and whole grains are beneficial in preventing MetS progression [12,14,25]. 

The diet profile identified in our research group was characterized by a higher frequency of consumption of red meat and meat products, sweets, sweetened beverages and non-wholegrain products. Similar results were obtained by Osadnik et al. [25], indicating that people with the MetS syndrome, compared to their metabolically healthy peers, were more likely to adhere the Western dietary pattern and had a poor-quality diet, regardless of anthropometric parameters such as BMI and WHR. This is consistent with the results of Fabiani et al. [26], who, in a systematic review and meta-analysis of 40 observational studies, showed that a high-fat diet, processed meat and sweets were significantly associated with an increased risk of MetS. Consumption of high glycemic index foods, such as cereals, confectionery, and sugar-sweetened beverages, is associated with a rapid release of carbohydrate, an increase in plasma glucose, and an increase in insulin secretion, leading to postprandial hyperinsulinemia, which has a lipogenic effect. As a result, this leads to insulin resistance, which is directly related to MetS [27]. The higher intake of whole grains seen in our study is associated with lower intakes of fiber and magnesium, which are also important in MetS.

In our study, the frequency of eating red meat and processed meat was high and above nutritional recommendations, and did not differ for form or number of MetS components. Meat, including processed meat, is one of the most popular food products in many European countries, and their higher consumption was related to higher income [28]. Additionally. in Poland [29], meat consumption is still a symbol of prestige and wealth; hence, the reduction of its consumption is difficult. 

We have not observed any effect of the frequency of eating more selected food group products and number of MetS components. Only in women, higher frequencies of eating sweets and “non-whole grains” products were found in sub-groups with more MetS components, while in men, the lower frequency of eating “milk and milk products” was found in men with five MetS components. Despite the fact that the Western diet should increase the risk of MetS, the results of many studies that have investigated this relationship have produced inconsistent results [30], as have our findings. A possible explanation for these inconsistencies could be the consideration of various lifestyle factors in the patterns created, including diet, physical activity, and smoking, as well as specifics of the study group (gender, ethnicity, size), and their possible synergistic effects on the odds of MetS.

Our data suggest that frequent consumption of fried foods may increase the severity of MetS, but after adjusting for gender, age, education, physical activity, and smoking, the effect lost statistical significance. It is unclear whether eating fried foods is associated with MetS severity. We know that frying increases the fat content of foods and can increase the concentration of trans fatty acids. In addition, frying inhibits the activity of paraoxonase, an enzyme that inhibits the oxidation of low-density lipoprotein (LDL)-cholesterol, and both oxidized LDL and trans fats have a proven effect in the pathogenesis of coronary artery disease, and this association is dose-dependent [31]. In addition, Kang and Kim [32] have shown significant association between fried food consumption and hypertension only in Korean women (in men it was not significant). In turn, the results of a recently published study showed that higher ultra-processed food (UPF) consumption was positively correlated with MetS, and the association was stronger in women, adults aged 45–59 and those living in urban areas [33]. This may be because UPFs are typically high in added sugars, salt, and saturated and trans fats; their excessive consumption may also result in an increase in the level of C-reactive protein (CRP) and intensification of inflammatory reactions, which increases the risk of MetS [34]. In addition, higher UPF intake is inversely related to a poor nutritional profile and the quality and deficiencies of dietary fiber, vegetables, fruits, and legumes, which may also be associated with a higher risk of MetS [35]. According to these results, we can recommend reducing fried food consumption and ultra-processed food, but further studies are needed to investigate the effect of different types of processed foods on MetS severity.

The results of this study confirmed that the frequency of fish consumption has a statistically significant relationship with the probability of severe MetS. It is results of the essential omega-3 polyunsaturated fatty acids (n-3 PUFAs) as eicosapentaenoic acid (EPA) and docosahexaenoic acid (DHA), which are mainly found in marine fish and algal oils and fatty fish. For the primary prevention of CVD, an intake of 250 mg EPA + DHA per day is recommended, which can be achieved by eating fish twice a week, including one serving of oily fish [36,37]. The proposed mechanisms explaining the action of n-3 PUFAs in the context of cardiometabolic changes include: reduction of plasma TG concentration [38,39], modulation of lipid metabolism, mainly through the regulating abilities of adipokines, such as adiponectin and leptin, as well as alleviation of inflammation by lowering pro-inflammatory cytokines IL-6, tumor necrosis factor alpha (TNF-α), as well as CRP in plasma, and promoting adipogenesis and changing epigenetic mechanisms [39].

Controversies still exist as to which anthropometric index best predicts cardiometabolic risk. In this present study, among anthropometric indices, the strongest correlation between BRI and MetS score was found. A possible explanation may be the fact that the BRI, compared to other anthropometric indices, estimates a human as an elliptical figure and somehow takes into account the general fat mass and visceral fat (VAT), which has a well-established association with MetS [40]. Several studies have similarly reported the superior power of BRI over the traditional anthropometric indices in predicting MetS [41,42,43]. Likewise, Anto at al. [44] indicated that only BRI was the independent predictor of MetS and compared to traditional indicators it turned out to be the best. Our study found that BRI had a strong positive correlation with WHtR and WC, and also with BMI and fat mass (%). Moreover, BRI had a stronger correlation than BMI and FM (%) with most cardiometabolic indices. The strong correlation with these indices supports and explains the observed superiority of BRI in predicting MetS severity. 

Hence, the results of this study provide evidence that diet, and especially dietary profile, may have an influence on the risk of severe MetS. Similarly, physical activity, which in our study group was declared low by 70% of respondents, despite the fact that in MetS therapy, regular and moderate physical activity is recommended. The combination of these factors, unhealthy diet and low physical activity, can act synergistically and lead to the further development of the disease. In fact, combining diet, food components, and exercise programs have been shown to reduce rates of MetS development and its components and maximize health effects [3,26,45,46].

### Strength and Limitations

The strength of our study is the relatively large homogeneous group with MetS treated in the Metabolic Diseases Outpatient Clinic. Second, in data analysis, we used different adjustment models to adjust potential confounding factors such as age, sex, education status, physical activity, smoking status and dietary patterns. Therefore, confounding factors were better controlled. Third, we applied multiple anthropometric and cardiometabolic indices simultaneously, including the VAI, AIP, CMI, LAP, TG/HDL, TyG-BMI, TyG-WC, BRI, WC, WHtR, BMI and FM, defined DPs and the number of MetS components. To the best of our knowledge, this was the first such study among adult Poles with metabolic disorders. Furthermore, the current study shows that the Body Roundness Index (BRI) should be recommended as the best predictor of cardiometabolic risk among adults with MetS in both women and men. 

Our study has several limitations. First, the study sample is not representative of the general population, but included people who came to the outpatient clinic with metabolic problems. Hence, it reflects those most at risk of MetS. Second, the cross-sectional design of the study does not consider causation. Data for each respondent were obtained only at one point of the study, but the many variables included in the analysis have great potential for developing a nutritional strategy for improving eating habits in adult populations in Poland. Third, we assessed consumption mainly in a qualitative context. We chose the validated FFQ because we wanted to see mainly dietary patterns in patients with metabolic disorders. In addition, using the FFQ, we were unable to assess the intake of nutrients such as antioxidant vitamins, fatty acids and dietary fiber, which affect the risk of MetS. In our analysis, we did not include supplements, only food products, although, e.g., EPA and DHA acids can also be supplied in supplements. We are aware of these limitations and plan to address them in the future. The obtained results, despite the limitations, are of practical importance and accurately reflect eating habits and existing dietary patterns, as well as suggest using BRI to fast diagnosis of cardiometabolic risk.

## 5. Conclusions

This study provided the necessary evidence to focus on “holistic” modification of life-style patterns in patients with MetS. Therefore, development of strategies to reduce the risk of more severe forms of MetS should be focused on teaching behaviors conducive to reducing fried and processed food consumption and, most of all, on to increasing fish consumption and other prohealthy food. Together with prohealthy diet, physical activity should also be included in the management of MetS. Public health policy should encourage the integration of lifestyle interventions into healthcare systems. Moreover, the results of this study demonstrated and confirmed the possibility of using Body Roundness Index to fast diagnosis of cardiometabolic risk.

## Figures and Tables

**Table 1:** Cut-off points for MetS diagnosis [3,5].

Parameter	Cut-Off Point for Men	Cut-Off Point for Women
WC	≥94 cm	≥80 cm
Glucose	≥100 mg/dL (≥5.56 mmol/L)	≥100 mg/dL (≥5.56 mmol/L)
Triglycerides	≥150 mg/dL (≥1.69 mmol/L)	≥150 mg/dL (≥1.69 mmol/L)
HDL-C	<40 mg/dL (<1.03 mmol/L)	<50 mg/dL (<1.29 mmol/L)
Blood pressure	SBP ≥ 130 or DBP ≥ 85 mmHg	SBP ≥ 130 or DBP ≥ 85 mmHg

WC, waist circumference; HDL-C, HDL cholesterol; SBP, systolic blood pressure; DBP, diastolic blood pressure.

**Table 2:** The characteristics of the participant group according to the number of MetS components.

Variable	Number of Metabolic Syndrome Criteria			
	3 MetS (*n* = 150)	4 MetS (*n* = 70)	5 MetS (*n* = 56)	*p*-Value
Age (years)	51.6 ± 13.2	54.9 ± 12.2	58.2 ± 9.6	0.004
Sex (%)				
men	22	69	64	<0.0001
women	78	31	36	
Education (%)				
primary and vocational	19	23	36	0.03
secondary	44	50	32	
university	37	27	32	
Physical activity (%)				
low	71	80	78	ns
moderate	25	19	20	
vigorous	4	1	2	
Smoking (%)	23	34	18	0.004
Anthropometric indices:				
BMI (%)				
<18.5	3.3	1.4	0	0.00015
18.5–24.99	31.3	8.6	3.6	
25.0–29.99	22.0	32.9	32.1	
30.0–34.99	24.0	27.1	25.0	
35.0–39.99	11.3	14.3	21.4	
>40	8.0	15.7	17.9	
BMI (kg/m^2^)	28.91 ± 6.91 28.15 ^a^	32.19 ± 6.41 31.41 ^b^	33.89 ± 6.71 33.09 ^b^	<0.0001
BRI	5.32 ± 2.20 5.24 ^a^	6.57 ± 1.98 6.21 ^b^	7.43 ± 2.16 7.35 ^b^	<0.0001
WC (cm)	97.24 ± 15.77 98.0 ^a^	111 ± 15.17 111.0 ^b^	115 ± 13.66 116.5 ^b^	<0.0001
WHtR	0.59 ± 0.10 0.59 ^a^	0.64 ± 0.08 0.63 ^b^	0.68 ± 0.08 0.68 ^b^	<0.0001
Fat mass (FM) (%)	33.92 ± 10.67 34.70	32.02 ± 9.43 30.75	33.81 ± 9.99 33.70	ns
Blood pressure:				
SBP (mmHg)	131 ± 18.07 126.00 ^a^	139 ± 16.84 139.50 ^b^	144 ± 14.99 141.00 ^b^	<0.0001
DBP (mmHg)	78.14 ± 12.28 80.00 ^a^	84.60 ± 11.24 85.00	84.82 ± 9.89 85.00	<0.0001
FPG (mmol/L)	6.62 ± 2.93 5.72 ^a^	7.68 ± 3.38 6.36 ^b^	8.31 ± 3.72 6.86 ^b^	<0.0001
Lipid profile:				
CHOL (mmol/L)	5.04 ± 0.86 5.22	5.01 ± 0.98 5.20	5.09 ± 0.99 5.22	ns
TG (mmol/L)	1.73 ± 0.53 1.73 ^a^	2.07 ± 0.85 1.81 ^b^	2.43 ± 0.73 2.13 ^c^	<0.0001
HDL-C (mmol/L)	1.27 ± 0.28 1.25 ^a^	1.01 ± 0.27 0.95 ^b^	0.86 ± 0.15 0.49 ^c^	<0.0001
LDL-C (mmol/L)	2.79 ± 0.77 2.72	2.72 ± 0.89 2.70	2.53 ± 0.78 2.50	ns
Cardiometabolic indices:				
AIP	1.12 ± 0.37 1.10 ^a^	1.50 ± 0.44 1.44 ^b^	1.83 ± 0.32 1.81 ^c^	<0.0001
CMI (mmol/L)	0.85 ± 0.40 0.74 ^a^	1.42 ± 0.81 1.19 ^b^	1.97 ± 0.78 1.77 ^c^	<0.0001
LAP (mmol/L)	66.24 ± 38.77 60.41 ^a^	101 ± 51.97 91.59 ^b^	130 ± 56.29 121.53 ^c^	<0.0001
TG/HDL-C ratio (mmol/L)	1.45 ± 0.61 1.33 ^a^	2.21 ± 1.27 1.86 ^b^	2.89 ± 1.00 2.70 ^c^	<0.0001
TyG	9.00 ± 0.44 8.89 ^a^	9.29 ± 0.47 9.15 ^ab^	9.56 ± 0.52 9.41 ^b^	<0.0001
TyG-BMI	260 ± 62.91 248 ^a^	299 ± 60.96 298 ^b^	324 ± 66.57 316 ^b^	<0.0001
TyG-WC	874 ± 146 878 ^a^	1035± 153 1062 ^b^	1105 ± 154 1093 ^b^	<0.0001
VAI (mmol/L)	2.64 ± 1.15 2.43 ^a^	3.58 ± 2.07 2.94 ^b^	4.80 ± 1.68 4.50 ^c^	<0.0001
Dietary patterns:				
Western	27	37	29	ns
Prudent	45	39	32	
Low Food	28	24	39	

AIP, atherogenic index of plasma; BMI, body mass index; BRI, body roundness index; CMI, cardiometabolic index CHOL, cholesterol; FM, fat mass; FPG, fasting plasma glucose; HDL-C, high-density lipoprotein; LDL-C, low-density lipoprotein; LAP, lipid accumulation product; SBP, systolic blood pressure; DBP, diastolic blood pressure; TG, triglyceride; TG/HDL-C, triglycerides to HDL-cholesterol ratio; TyG, triglycerides–glucose index; TyG–BMI, TyG–body mass index; TyG–WC, TyG–waist circumference index; WC, waist circumference; WHR, waist-to-hip ratio; WHtR, waist-to-height ratio; VAI, visceral adiposity index; ns, not significant; different letters indicate that the samples are significantly different at *p* < 0.05.

**Table 3:** Dietary patterns and frequency of consumption of selected group products by sex and number of MetS components.

Variable	Men (*n* = 117)			*p*-Value	Women (*n* = 159)			*p*-Value	*p*-Value Men vs. Women		
	3 MetS (*n* = 33)	4 MetS (*n* = 48)	5 MetS (*n* = 36)		3 MetS (*n* = 117)	4 MetS (*n* = 20)	5 MetS (*n* = 22)		3 MetS	4 MetS	5 MetS
Dietary patterns (%)											
Western	36	31	28	ns	24	50	30	0.077	ns		
Prudent	39	40	33		46	36	30		ns		
Low Food	25	29	39		30	14	40		ns		
Food groups ^#^ (Mean ± SD)											
Vegetable	5.5 ± 1.1	5.3 ± 1.4	4.9 ± 1.5	ns	5.6 ± 1.0	5.5 ± 1.0	5.5 ± 0.9	ns	ns	ns	ns
Fruit	6.0 ± 0.8	5.7 ± 1.2	5.9 ± 0.8	ns	5.9 ± 0.9	5.9 ± 0.8	6.3 ± 0.9	ns	ns	ns	0.095
Milk, fermented milk beverages, cottage cheese	5.1 ± 1.0	4.4 ± 1.4	4.1 ± 1.4	0.007	4.8 ± 1.3	5.1 ± 0.9	4.6 ± 1.7	ns	ns	0.081	ns
Cheese	4.1 ± 1.2	4.0 ± 1.3	3.9 ± 1.1	ns	3.7 ± 1.2	3.6 ± 1.2	3.7 ± 1.2	ns	0.073	ns	ns
Fish	3.0 ± 0.8	2.6 ± 0.8	2.7 ± 0.7	ns	2.7 ± 0.7	2.6 ± 0.7	2.6 ± 0.7	ns	ns	ns	ns
Red meat	3.9 ± 0.7	4.1 ± 0.8	4.2 ± 0.7	ns	3.9 ± 0.8	3.8 ± 0.8	3.7 ± 0.8	ns	ns	ns	ns
White meat	3.9 ± 0.7	3.9 ± 0.9	3.9 ± 0.8	ns	4.1 ± 0.8	3.9 ± 0.6	3.9 ± 1.1	ns	ns	ns	ns
Processed meat	5.9 ± 1.0	5.9 ± 1.1	5.9 ± 1.1	ns	5.7 ± 1.0	5.7 ± 1.1	5.9 ± 1.0	ns	ns	ns	ns
Sweets	4.0 ± 1.8	3.7 ± 1.6	3.5 ± 1.9	ns	3.9 ± 1.6	4.1 ± 1.5	2.9 ± 1.3	0.007	ns	ns	ns
Whole grains	3.9 ± 1.9	3.9 ± 1.8	4.3 ± 1.8	ns	4.2 ± 1.9	4.2 ± 1.7	3.9 ± 2.1	ns	ns	ns	ns
Non-whole grains	6.0 ± 1.3	5.9 ± 1.5	6.0 ± 1.4	ns	5.8 ± 1.3	6.44 ± 0.7	6.2 ± 1.2	0.045	ns	ns	ns
Fried foods	4.1 ± 1.4	4.2 ± 1.2	4.1 ± 1.3	ns	3.5 ± 1.3	3.8 ± 1.2	4.3 ± 1.6	ns	0.048	ns	0.074
Fast food	2.4 ± 1.1	2.0 ± 0.8	2.1 ± 1.0	ns	1.8 ± 0.7	1.9 ± 0.9	1.9 ± 0.7	ns	0.008	ns	ns
Water	5.6 ± 0.7	5.4 ± 1.2	5.7 ± 0.8	ns	5.6 ± 0.8	5.6 ± 0.7	5.9 ± 0.5	ns	ns	ns	ns
Juices	3.7 ± 1.5	3.9 ± 1.6	4.1 ± 1.6	ns	4.1 ± 1.5	4.1 ± 1.4	4.4 ± 1.7	ns	ns	ns	ns
Sweet beverages	3.7 ± 1.8	3.5 ± 1.8	4.1 ± 1.6	ns	3.0 ± 1.5	2.8 ± 1.3	2.6 ± 1.4	ns	ns	ns	0.001
Coffee and tea	6.4 ± 1.6	6.1 ± 1.9	6.3 ± 1.7	ns	6.5 ± 1.5	6.7 ± 1.3	6.8 ± 0.7	ns	ns	ns	ns
Energy drinks	1.4 ± 0.9	1.3 ± 0.7	1.2 ± 0.5	ns	1.2 ± 0.5	1.2 ± 0.5	1.1 ± 0.2	ns	ns	ns	ns

MetS, Metabolic Syndrome; ^#^ results are expressed in number of times a week for each food group; ns, not significant.

**Table 4:** Anthropometric parameters, cardiometabolic indices and number of MetS components.

Variable	Men (*n* = 117)			*p*-Value	Women (*n* = 159)			*p*-Value	*p*-Value Men vs. Women		
	3 MetS (*n* = 33)	4 MetS (*n* = 48)	5 MetS (*n* = 36)		3 MetS (*n* = 117)	4 MetS (*n* = 20)	5 MetS (*n* = 22)		3 MetS	4 MetS	5 MetS
Anthropometric indices:											
BMI (kg/m^2^)	26.9 ± 5.2 26.5 ^a^	31.70 ± 5.6 31.4 ^b^	32.7 ± 5.1 31.1 ^b^	<0.0001	29.5 ± 7.2 29.3 ^a^	33.3 ± 7.9 32.1 ^ab^	36.0 ± 8.7 36.3 ^b^	0.002	ns	ns	ns
BRI	4.7 ± 1.7 4.4 ^a^	6.5 ± 1.9 6.0 ^b^	7.0 ± 1.7 6.7 ^b^	<0.0001	5.5 ± 2.3 5.4 ^a^	6.8 ± 2.2 7.2 ^ab^	8.3 ± 2.7 8.6 ^b^	<0.0001	ns	ns	0.042
WC (cm)	99.4 ± 14.8 97.0 ^a^	113.8 ± 14.5 112.5 ^b^	116.6 ± 12.8 116.5 ^b^	<0.0001	96.6 ± 16.0 98.5 ^a^	106 ± 15.6 107.5 ^b^	114 ± 15.2 116.0 ^b^	<0.0001	ns	ns	ns
WHtR	0.56 ± 0.08 0.55 ^a^	0.64 ± 0.08 0.63 ^b^	0.66 ± 0.07 0.65 ^b^	<0.0001	0.59 ± 0.10 0.60 ^a^	0.65 ± 0.09 0.67 ^ab^	0.71 ± 0.10 0.73 ^b^	<0.0001	ns	ns	0.042
FM (%)	23.2 ± 9.2 24.2 ^a^	28.1 ± 7.0 28.3 ^b^	29.2 ± 7.4 29.0 ^b^	0.007	36.9 ± 9.0 38.2 ^a^	40.5 ± 8.6 41.1 ^ab^	42.1 ± 8.8 45.1 ^b^	0.019	0.000	0.000	0.000
Blood pressure:											
SBP (mmHg)	129 ± 16.2 128 ^a^	138 ± 17.5 140 ^b^	141 ± 14.6 140 ^b^	0.005	132 ± 18.6 126 ^a^	140 ± 15.7 139 ^b^	148 ± 14.8 145 ^b^	<0.0001	ns	ns	ns
DBP (mmHg)	76.5 ± 12.1 78.0 ^a^	85.5 ± 11.9 85.0 ^b^	86.1 ± 8.6 85.0 ^b^	0.001	78.6 ± 12.3 80.0	82.5 ±9.7 83.5	82.5 ± 11.8 85.0	ns	ns	ns	ns
FPG (mmol/L)	6.43 ± 2.84 5.4 ^a^	8.03 ± 3.71 6.4 ^b^	8.52 ±3.93 6.9 ^b^	0.0006	6.67 ± 2.97 5.75 ^a^	6.92 ± 2.44 6.36 ^ab^	7.92 ± 3.34 6.78 ^b^	0.006	ns	ns	ns
Lipid profile:											
CHOL (mmol/L)	5.1 ± 0.9 5.2	4.9 ± 1.0 5.1	5.2 ± 1.1 5.2	ns	5.0 ± 0.9 5.2	5.2 ± 0.8 5.3	4.9 ± 0.8 5.2	ns	ns	ns	ns
TG (mmol/L)	1.7 ± 0.5 1.7 ^a^	2.1 ±1.0 1.8 ^a^	2.5 ± 0.8 2.1 ^b^	<0.0001	1.7 ± 0.5 1.7 ^a^	2.0 ± 0.5 2.0 ^b^	2.2 ± 0.5 2.1 ^b^	<0.0001	ns	ns	ns
HDL-C (mmol/L)	1.2 ± 0.3 1.1 ^a^	1.0 ± 0.3 0.9 ^b^	0.9 ± 0.2 0.9 ^b^	<0.0001	1.3 ± 0.3 1.3 ^a^	1.1 ± 0.3 1.1 ^b^	0.9 ± 0.1 0.9 ^c^	<0.0001	0.014	0.019	ns
LDL-C (mmol/L)	2.8 ± 0.7 2.8	2.7 ± 0.9 2.6	2.5 ± 0.8 2.4	ns	2.8 ± 0.8 2.7	2.8 ± 0.8 2.8	2.5 ± 0.7 2.7 ^c^	ns	ns	ns	ns
Cardiometabolic indices:											
AIP	1.2 ± 0.4 1.2 ^a^	1.5 ± 0.5 1.5 ^b^	1.9 ± 0.3 1.8 ^c^	<0.0001	1.1 ± 0.4 1.1 ^a^	1.4 ± 0.4 1.4 ^b^	1.8 ± 0.3 1.7 ^c^	<0.0001	ns	ns	ns
CMI (mmol/L)	0.9 ± 0.5 0.8 ^a^	1.5 ± 0.9 1.2 ^b^	2.0 ± 0.9 1.8 ^c^	<0.0001	0.8 ± 0.4 0.7 ^a^	1.3 ± 0.5 1.1 ^b^	1.9 ± 0.6 1.7 ^c^	<0.0001	ns	ns	ns
LAP (mmol/L)	59.3 ± 40.2 48.6 ^a^	102 ± 55.1 89.5 ^b^	134 ± 62.2 127.4 ^c^	<0.0001	68.2 ± 38.3 64.2 ^a^	99.4 ± 45.6 96.9 ^b^	123 ± 44.4 119 ^b^	<0.0001	ns	ns	ns
TG/HDL-C ratio (mmol/L)	1.6 ± 0.7 1.4 ^a^	2.3 ± 1.4 1.9 ^b^	3.0 ± 1.1 2.8 ^c^	<0.0001	1.4 ± 0.6 1.3 ^a^	1.9 ± 0.8 1.8 ^b^	2.7 ± 0.7 2.4 ^c^	<0.0001	ns	ns	ns
TyG	8.9 ± 0.4 8.8 ^a^	9.3 ± 0.5 9.2 ^ab^	9.6 ± 0.5 9.4 ^b^	<0.0001	9.0 ± 0.4 8.9 ^a^	9.2 ± 0.4 9.1 ^b^	9.5 ± 0.5 9.3 ^b^	<0.0001	ns	ns	ns
TyG-BMI	240 ± 47.7 225 ^a^	295 ± 53.7 294 ^b^	315.± 55.7 295 ^b^	<0.0001	266 ± 65.7 261 ^a^	308 ± 75.0 303 ^ab^	340 ± 81.9 344 ^b^	<0.0001	ns	ns	ns
TyG-WC	888 ± 135 866 ^a^	1061 ± 146 1069 ^b^	1123 ± 156 1105 ^b^	<0.0001	871 ± 150 880 ^a^	982 ± 159 992 ^b^	1072 ± 148 1091 ^b^	<0.0001	ns	ns	ns
VAI (mmol/L)	2.2 ± 1.0 2.0 ^a^	3.4 ± 2.2 2.8 ^b^	4.4 ± 1.7 4.2 ^c^	<0.0001	2.8 ± 1.2 2.6 ^a^	3.9 ± 1.7 3.4 ^b^	5.4 ± 1.5 5.1 ^c^	<0.0001	0.003	0.049	0.013

AIP, atherogenic index of plasma; BMI, body mass index; BRI, body roundness index; CMI, car-diometabolic index CHOL, cholesterol; FM, fat mass; FPG, fasting plasma glucose; HDL-C, high-density lipoprotein; LDL-C, low-density lipoprotein; LAP, lipid accumulation product; SBP, systolic blood pressure; DBP, diastolic blood pressure; TG, triglyceride; TG/HDL-C, triglycerides to HDL-cholesterol ratio; TyG, triglycerides–glucose index; TyG–BMI, TyG–body mass index; TyG–WC, TyG–waist circumference index; WC, waist circumference; WHR, waist-to-hip ratio; WHtR, waist-to-height ratio; VAI, visceral adiposity index; ns, not significant; different letters indicate that the samples are significantly different at *p* < 0.05.

**Table 5:** The partial coefficients of Spearman correlation between analyzed variables in patients with MetS, adjusted on age and sex.

Variables	WC	WHR	WHtR	BMI	FM (%)	BRI	TyG	CHOL	HDL-C	TG	LDL-C	MetS Score
Anthropometric indices:												
BMI (kg/m^2^)	0.889 ***	0.375 **	0.900 ***	-	0.830 ***	0.899 ***	0.110	0.117	−0.135 *	0.181 *	0.098	0.324 **
BRI	0.958 ***	0.600 **	0.995 ***	0.899 ***	0.789 ***	-	0.174 *	0.079	−0.183 *	0.215 *	0.032	0.376 **
FM (%)	0.804 ***	0.380 **	0.802 ***	0.830 ***	-	0.788 ***	0.064	0.132 *	−0.029	0.182 *	0.094	0.214 *
WC	-	0.629 ***	0.964 ***	0.889 ***	0.804 ***	0.985 ***	0.165 *	0.105	−0.166 *	0.236 *	0.080	0.363 **
WHR	0.629 ***	-	0.608 ***	0.375 **	0.380 **	0.598 **	0.178 *	0.107	−0.106	0.162 *	0.085	0.214 *
WHtR	0.964 ***	0.608 ***	-	0.900 ***	0.802 ***	0.995 ***	0.164 *	0.090	−0.168 *	0.218 *	0.044	0.366 **
Lipid profile:												
CHOL (mmol/L)	0.105	0.107	0.090	0.117	0.132 *	0.080	0.269 *	-	0.241 *	0.369 **	0.690 ***	0.025
HDL-C (mmol/l)	−0.166 *	−0.106	−0.168 *	−0.135 *	−0.029	−0.182 *	−0.213 *	0.241 *	-	−0.201 *	0.108	−0.458 **
TG (mmol/L)	0.236 *	0.162 *	0.218 *	0.181 *	0.182 *	0.217 *	0.703 ***	0.369 **	−0.201 *	-	0.145 *	0.364 **
LDL-C (mmol/L)	0.080	0.085	0.044	0.098	0.094	0.084	0.084	0.690 ***	0.108	0.145 *	-	−0.088
TyG	0.165 *	0.178 *	0.164 *	0.110	0.064	0.174 *	-	0.269	−0.213 *	0.703 ***	0.084	0.402 **
MetS score	0.363 **	0.214 *	0.366 **	0.324 **	0.214 *	0.376 **	0.402 **	0.025	−0.458 **	0.364 **	−0.088	-
Cardiometabolic indices:												
AIP	0.273 *	0.180 *	0.264 *	0.212 *	0.145 *	0.274 *	0.641 ***	0.143 *	−0.701 ***	0.817 ***	0.039	0.548 **
CMI (mmol/L)	0.472 **	0.307 **	0.464 **	0.393 **	0.335 **	0.473 **	0.597 **	0.192 *	−0.570 **	0.814 ***	0.064	0.540 **
LAP (mmol/L)	0.753 ***	0.473 **	0.716 ***	0.651 ***	0.593 **	0.716 ***	0.548 **	0.317 **	−0.216 *	0.792 ***	0.143 *	0.460 **
TG/HDL (mmol/L)	0.251 *	0.178 *	0.237 *	0.184 *	0.145 *	0.244 *	0.625 ***	0.183 *	−0.595 **	0.847 ***	0.066	0.484 **
TyG-BMI	0.884 ***	0.401 **	0.892 ***	0.974 ***	0.805 ***	0.894 ***	0.329 *	0.178 *	−0.173 *	0.333 **	0.114	0.403 **
TyG-WC	0.949 ***	0.624 ***	0.915 ***	0.829 ***	0.743 ***	0.913 ***	0.465 **	0.188 *	−0.214 **	0.438 **	0.103	0.457 **
VAI (mmol/L)	0.312 **	0.258 *	0.288 *	0.190 *	0.161 *	0.293 *	0.618 ***	0.173 *	−0.612 ***	0.829 ***	0.069	0.504 **

* *p* ≤ 0.05; ** *p* ≤ 0.01; *** *p* ≤ 0.001.

**Table 6:** Results of logistic regression analysis between MetS score 4–5 and selected nutritional variables.

Variable	Univariate Model 1	Multivariate Model 2	Multivariate Model 3
	OR (95% CI)	aOR (95% CI)	aOR (95% CI)
Fried foods:			
Never or almost never	Ref.	Ref.	Ref.
At least one time per week	2.13 (1.00–4.54) *	1.43 (0.60–3.39)	1.36 (0.56–3.32)
At least one time per day	3.37 (1.30–8.74) **	1.92 (0.64–5.77)	1.75 (0.55–5.56)
Fish intake:			
Never or almost never	1.35 (0.82–2.22) *	2.03 (1.10–3.74) *	1.98 (1.07–3.69) *
At least one time per week	Ref.	Ref.	Ref.
Dietary patterns:			
Western	1.56 (0.88–2.78)	1.51 (0.76–3.01)	
Prudent	Ref.	Ref.	-
Low Food	1.35 (0.76–2.40)	1.30 (0.63–2.71)	

aOR, adjusted odds ratio; CI, confidence intervals; OR, odds ratio; Model 2 adjusted for sex, age, education status, physical activity and smoking status; Model 3: model 2 adjusted for dietary pattern; * *p* ≤ 0.05; ** *p* ≤ 0.01.

## Data Availability

The data presented in this study are available on request from the corresponding author.

## Supplementary Materials

The following supporting information can be downloaded at: https://www.mdpi.com/article/10.3390/nu15102237/s1, Table S1. Dietary patterns description.

## References

[1] World Health Organization (2022). WHO European Regional Obesity Report 2022.

[2] The International Diabetes Federation IDF Consensus Worldwide Definition of the Metabolic Syndrome. https://www.idf.org/e-library/consensus-statements/60-idfconsensus-worldwide-definitionof-the-metabolic-syndrome.html.

[3] Ambroselli D., Masciulli F., Romano E., Catanzaro G., Besharat Z.M., Massari M.C., Ferretti E., Migliaccio S., Izzo L., Ritieni A. (2023). New Advances in Metabolic Syndrome, from Prevention to Treatment: The Role of Diet and Food. Nutrients.

[4] Ross R., Neeland I.J., Yamashita S., Shai I., Seidell J., Magni P., Santos R.D., Arsenault B., Cuevas A., Hu F.B. (2020). Waist circumference as a vital sign in clinical practice: A Consensus Statement from the IAS and ICCR Working Group on Visceral Obesity. Nat. Rev. Endocrinol..

[5] Alberti K.G.M.M., Eckel R.H., Grundy S.M., Zimmet P.Z., Cleeman J.I., Donato K.A., Fruchart J.C., James W.P.T., Loria C.M., Smith S.C. (2009). Harmonizing the metabolic syndrome: A joint interim statement of the international diabetes federation task force on epidemiology and prevention; National heart, lung, and blood institute; American heart association; World heart federation; International. Circulation.

[6] Pluta W., Dudzińska W., Lubkowska A. (2022). Metabolic Obesity in People with Normal Body Weight (MONW)—Review of Diagnostic Criteria. Int. J. Environ. Res. Public Health.

[7] Moore J.X., Chaudhary N., Akinyemiju T. (2017). Metabolic Syndrome Prevalence by Race/Ethnicity and Sex in the United States, National Health and Nutrition Examination Survey, 1988–2012. Prev. Chronic. Dis..

[8] Scuteri A., Laurent S., Cucca F., Cockcroft J., Cunha P.G., Mañas L.R., Raso F.U.M., Muiesan M.L., Ryliškyte L., Rietzschel E. (2015). Metabolic Syndrome and Arteries Research (MARE) Consortium. Metabolic syndrome across Europe: Different clusters of risk factors. Eur. J. Prev. Cardiol..

[9] GBD 2017 Causes of Death Collaborators (2018). Global, regional, and national age-sex-specific mortality for 282 causes of death in 195 countries and territories, 1980–2017: A systematic analysis for the Global Burden of Disease Study 2017. Lancet.

[10] Saklayen M.G. (2018). The Global Epidemic of the Metabolic Syndrome. Curr. Hypertens. Rep..

[11] Regufe V.M.G., Pinto C.M.C.B., Perez P.M.V.H. (2020). Metabolic syndrome in type 2 diabetic patients: A review of current evidence. Porto Biomed. J..

[12] de la Iglesia R., Loria-Kohen V., Zulet M.A., Martinez J.A., Reglero G., de Molina A.R. (2016). Dietary Strategies Implicated in the Prevention and Treatment of Metabolic Syndrome. Int. J. Mol. Sci..

[13] McCracken E., Monaghan M., Sreenivasan S. (2018). Pathophysiology of the metabolic syndrome. Clin. Dermatol..

[14] Agodi A., Maugeri A., Kunzova S., Sochor O., Bauerova H., Kiacova N., Barchitta M., Vinciguerra M. (2018). Association of Dietary Patterns with Metabolic Syndrome: Results from the Kardiovize Brno 2030 Study. Nutrients.

[15] ISAK (2001). International Standards for Anthropometric Assessment; International Society for the Advancement of Kinanthropometry: Potchefstroom. http://www.ceap.br/material/MAT17032011184632.pdf.

[16] Stewart A., Marfell-Jones M.J., International Society for the Advancement of Kinanthropometry (2011). International Standards for Anthropometric Assessment.

[17] Holmes C.J., Racette S.B. (2021). The Utility of Body Composition Assessment in Nutrition and Clinical Practice: An Overview of Current Methodology. Nutrients.

[18] Górnicka M., Szewczyk K., Białkowska A., Jancichova K., Habanova M., Górnicki K., Hamulka J. (2022). Anthropometric Indices as Predictive Screening Tools for Obesity in Adults; The Need to Define Sex-Specific Cut-Off Points for Anthropometric Indices. Appl. Sci..

[19] Lee D.H., Keum N., Hu F.B., Orav E., Rimm E., Sun Q., Willett W.C., Giovannucci E. (2017). Development and validation of anthropometric prediction equations for lean body mass, fat mass and percent fat in adults using the National Health and Nutrition Examination Survey (NHANES) 1999–2006. Br. J. Nutr..

[20] National Institute for Health and Care Excellence (NICE) Hypertension in Adults: Diagnosis and Management. https://www.nice.org.uk/guidance/ng136.

[21] Friedewald W.T., Levy R.I., Fredrickson D.S. (1972). Estimation of the Concentration of Low-Density Lipoprotein Cholesterol in Plasma, Without Use of the Preparative Ultracentrifuge. Clin. Chem..

[22] Rodríguez-Carrillo P.L., Aguirre-Tostado P.I., Macías-Cervantes M.H., Alegría-Torres J.A., Luevano-Contreras C. (2021). Novel Adiposity and Biochemical-Anthropometric Indices to Identify Cardiometabolic Risk and Metabolic Syndrome in Mexican Adults. Healthcare.

[23] (2014). Dietary Habits and Nutrition Beliefs Questionnaire and the Manual for Developing of Nutritional Data KomPAN [in Polish: Kwestionariusz do Badania Poglądów i Zwyczajów Żywieniowych Oraz Procedura Opracowania Danych (KomPAN^®^): Wersja Polskojęzyczna. http://www.knozc.pan.pl/.

[24] Kowalkowska J., Wadolowska L., Czarnocinska J., Czlapka-Matyasik M., Galinski G., Jezewska-Zychowicz M., Bronkowska M., Dlugosz A., Loboda D., Wyka J. (2018). Reproducibility of a Questionnaire for Dietary Habits, Lifestyle and Nutrition Knowledge Assessment (KomPAN) in Polish Adolescents and Adults. Nutrients.

[25] Osadnik K., Osadnik T., Lonnie M., Lejawa M., Reguła R., Fronczek M., Gawlita M., Wądołowska L., Gąsior M., Pawlas N. (2020). Metabolically healthy obese and metabolic syndrome of the lean: The importance of diet quality. Analysis of MAGNETIC cohort. Nutr. J..

[26] Fabiani R., Naldini G., Chiavarini M. (2019). Dietary Patterns and Metabolic Syndrome in Adult Subjects: A Systematic Review and Meta-Analysis. Nutrients.

[27] Gierach M., Junik R. (2021). Insulin resistance in metabolic syndrome depending on the occurrence of its components. Endokrynol. Pol..

[28] Mata J., Kadel P., Frank R., Schüz B. (2023). Education- and income-related differences in processed meat consumption across Europe: The role of food-related attitudes. Appetite.

[29] Jezewska-Zychowicz M., Gębski J., Plichta M., Guzek D., Kosicka-Gębska M. (2019). Diet-Related Factors, Physical Activity, and Weight Status in Polish Adults. Nutrients.

[30] Al Thani M., Al Thani A.A., Al-Chetachi W., Al Malki B., Khalifa S.A.H., Bakri A.H., Hwalla N., Nasreddine L., Naja F. (2016). A ‘High Risk’ Lifestyle Pattern Is Associated with Metabolic Syndrome among Qatari Women of Reproductive Age: A Cross-Sectional National Study. Int. J. Mol. Sci..

[31] Honerlaw J.P., Ho Y.L., Nguyen X.M.T. (2020). Fried food consumption and risk of coronary artery disease: The Million Veteran Program. Clin. Nutr..

[32] Kang Y., Kim J. (2016). Association between fried food consumption and hypertension in Korean adults. Br. J. Nutr..

[33] Pan F., Wang Z., Wang H., Zhang J., Su C., Jia X., Du W., Jiang H., Li W., Wang L. (2023). Association between Ultra-Processed Food Consumption and Metabolic Syndrome among Adults in China—Results from the China Health and Nutrition Survey. Nutrients.

[34] Christ A., Lauterbach M., Latz E. (2019). Western Diet and the Immune System: An Inflammatory Connection. Immunity.

[35] Batal M., Johnson-Down L., Moubarac J.C., Ing A., Fediuk K., Sadik T., Tikhonov C., Chan L., Willows N. (2018). Quantifying associations of the dietary share of ultra-processed foods with overall diet quality in First Nations peoples in the Canadian provinces of British Columbia, Alberta, Manitoba and Ontario. Public Health Nutr..

[36] US Departament of Health and Human Services Dietary Guidelines for Americans. https://health.gov/our-work/nutrition-physical-activity/dietary-guidelines.

[37] Jarosz M., Rychlik E., Stoś K., Charzewska J. (2020). Polish Dietary Reference Intakes—Revision.

[38] Khan S.U., Lone A.N., Khan M.S., Virani S.S., Blumenthal R., Nasir K., Miller M., Michos E.D., Ballantyne C.M., Boden W.E. (2021). Effect of omega-3 fatty acids on cardiovascular outcomes: A systematic review and meta-analysis. E Clin. Med..

[39] Albracht-Schulte K., Kalupahana N.S., Ramalingam L., Wang S., Rahman S.M., Robert-McComb J. (2018). Omega-3 fatty acids in obesity and metabolic syndrome: A mechanistic update. J. Nutr. Biochem..

[40] Lee J.J., Pedley A., Hoffmann U., Massaro J.M., Fox C.S. (2016). Association of Changes in Abdominal Fat Quantity and Quality With Incident Cardiovascular Disease Risk Factors. J. Am. Coll. Cardiol..

[41] Tian S., Zhang X., Xu Y., Dong H. (2016). Feasibility of body roundness index for identifying a clustering of cardiometabolic abnormalities compared to BMI, waist circumference and other anthropometric indices: The China Health and Nutrition Survey, 2008 to 2009. Medicine.

[42] Li G., Wu H.K., Wu X.W., Cao Z., Tu Y.C., Ma Y., Li B.N., Peng Q.Y., Cheng J., Wu B. (2019). The feasibility of two anthropometric indices to identify metabolic syndrome, insulin resistance and inflammatory factors in obese and overweight adults. Nutrition.

[43] Rico-Martín S., Calderón-García J.F., Sánchez-Rey P., Franco-Antonio C., Martínez Alvarez M., Sánchez Muñoz-Torrero J.F. (2020). Effectiveness of body roundness index in predicting metabolic syndrome: A systematic review and meta-analysis. Obes. Rev..

[44] Anto E.O., Frimpong J., Ivy W., Boadu O. (2022). Prevalence of Cardiometabolic Syndrome and its Association with Body Shape Index and A Body Roundness Index Among Type 2 Diabetes Mellitus Patients: A Hospital-Based Cross-Sectional Study in a Ghanaian Population. Front. Clin. Diabetes Healthc..

[45] Andersen C.J., Fernandez M.L. (2013). Dietary strategies to reduce metabolic syndrome. Rev. Endocr. Metab. Disord..

[46] Pérez-Martínez P., Mikhailidis D.P., Athyros V.G., Bullo M., Couture P., Covas M.I., de Koning L., Delgado-Lista J., Díaz-López A., Drevon C.A. (2017). Lifestyle recommendations for the prevention and management of metabolic syndrome: An international panel recommendation. Nutr. Rev..

