# Supplementary material for: Associations between Dietary Patterns, Anthropometric and Cardiometabolic Indices and the Number of MetS Components in Polish Adults with Metabolic Disorders
Source: Nutrients. 2023 May 9;15(10):2237. doi: 10.3390/nu15102237 (PMC10224261; doi:10.3390/nu15102237)
Supplement: Supplementary file 1 [file nutrients-15-02237-s001.zip › nutrients-2373804-supplementary.pdf]

nutrients

MDPI

Article

Associations between Dietary Patterns, Anthropometric and Cardiometabolic Indices and the number of MetS components in Polish Adults with Metabolic Disorders

Table S1. Dietary patterns description.

Variable	Western n=82 (29.7%)	Prudent n=112 (40.6%)	Low Food n=82 (29.7%)	p-value
Vegetable	5.6 ± 1.0	5.8 ± 0.9	4.7 ± 1.4	<0.0001
Fruit	6.1 ± 0.8	6.0 ± 0.9	5.6 ± 1.0	0.006
Milk, fermented milk beverages, cottage cheese	4.9 ± 1.2	5.1 ± 1.1	2.8 ± 1.3	<0.0001
Cheese	4.5 ± 1.1	3.7 ± 1.1	3.2 ± 1.1	<0.0001
Fish	2.7 ± 0.7	2.8 ± 0.7	2.6 ± 0.8	0.024
Red meat	4.0 ± 0.8	3.9 ± 0.8	4.0 ± 0.8	0.578
White meat	4.0 ± 0.8	4.2 ± 0.6	3.7 ± 1.0	0.001
Processed meat	6.2 ± 0.8	5.7 ± 1.1	5.5 ± 1.1	<0.0001
Sweets	5.1 ± 1.6	3.4 ± 1.4	2.9 ± 1.2	<0.0001
Whole grains	3.4 ±1.4	5.7 ± 1.0	2.6 ± 1.2	<0.0001
Non-whole grains	6.6 ± 0.8	5.3 ± 1.6	6.2 ± 0.9	<0.0001
Fried foods	4.8 ± 1.1	3.4 ± 1.2	3.5 ± 1.2	<0.0001
Fast-food	2.4 ± 0.8	1.7 ± 0.7	1.8 ± 0.8	<0.0001
Water	5.5 ± 0.7	5.7 ± 0.8	5.5 ± 1.0	0.085
Juices	4.5 ± 1.5	3.7 ± 1.5	4.1 ± 1.6	0.055
Sweet beverages	4.0 ±1.6	2.7 ± 1.5	3.3 ± 1.6	<0.0001
Coffee and tea	6.6 ± 1.2	6.6 ± 1.3	6.0 ± 2.0	0.168
Energy drinks	1.2 ± 0.5	1.2 ± 0.6	1.2 ± 0.6	0.994

Results are expressed in number of times a week for each food group.
